# Case Report: An unusual case of hemoptysis

**DOI:** 10.3389/fmed.2025.1549973

**Published:** 2025-04-03

**Authors:** Zhenhua Li, Zhen Yang, Jixiang Ni, Siyi Tang, Dongping Xia, Rujuan Wang

**Affiliations:** Department of Respiratory and Critical Care Medicine, The Central Hospital of Wuhan, Tongji Medical College, Huazhong University of Science and Technology, Wuhan, China

**Keywords:** hemoptysis, thyrocervical trunk, pseudoaneurysm, endovascular coiling, case report

## Abstract

We present a case of a 61-year-old woman with hemoptysis for 1 week. Two weeks prior, she underwent trigeminal microvascular decompression and repair of cerebrospinal fluid leakage under general anesthesia. Hemoptysis began 1 week after surgery, and examinations indicated intratracheal pseudoaneurysm. A definitive diagnostic evaluation confirmed that the formation of intratracheal pseudoaneurysm was related to tracheal intubation. This case highlights the fatal risk resulting from tracheal intubation. The patient was discovered after endovascular coiling. The utilization of endovascular approaches has gained popularity due to their minimally invasive nature, feasibility, and enhanced safety profile with reduced complications.

## Introduction

Pseudoaneurysm of the thyrocervical trunk is an exceedingly rare complication of trauma to the neck and can be life-threatening. Most cases of pseudoaneurysm are iatrogenic in nature and occur as a result of attempted cannulation of the internal jugular or subclavian veins for central venous access (1). Post-intubation tracheal injury is a rare complication associated with general anesthesia. If not promptly treated, it often leads to severe complications and even death. Iatrogenic damage is an increasingly common mechanism of tracheal injury (2). Injuries resulting from endotracheal intubation are likely the most common of the iatrogenic injuries and therefore require further comment.

## Case description

We describe a 61-year-old woman who presented with hemoptysis for 1 week, characterized by bright red blood in the sputum, with an estimated volume of 5–10 mL per day. Two weeks ago, she underwent trigeminal microvascular decompression and repair of cerebrospinal fluid leakage under general anesthesia at the other hospital. She had no fever, cough, phlegm, chest pain, difficulty breathing, or weight loss. She did not have abdominal pain, vomiting, or diarrhea. She was treated with antibiotics for 1 week, and the symptoms had not abated. The patient is retired and lives in an apartment in an urban area of the central region of China with her partner and their daughter. She reported no recent travel. She had no history of smoking, drinking, or drug abuse.

On admission, her temperature was 36.2°C, BP was 115/69 mm Hg, heart rate was 87/min, respiratory rate was 18 breaths per min, and peripheral oxygen saturation was 97% on room air. Both lungs were ausculted with clear breath sounds. Physical examination upon arrival was otherwise unremarkable.

The laboratory data were as follows: Laboratory investigations revealed normal findings for complete blood count (CBC), hepatic and renal function, electrolytes, cardiac enzyme profile, and coagulation studies. The respiratory syncytial virus, SARS-CoV-2, and influenza virus A/B were all negative. Antimicrobial therapy and intravenous infusion of snake venom hemagglutinin were taken. However, symptoms of hemoptysis did not improve.

**Figure 1:**
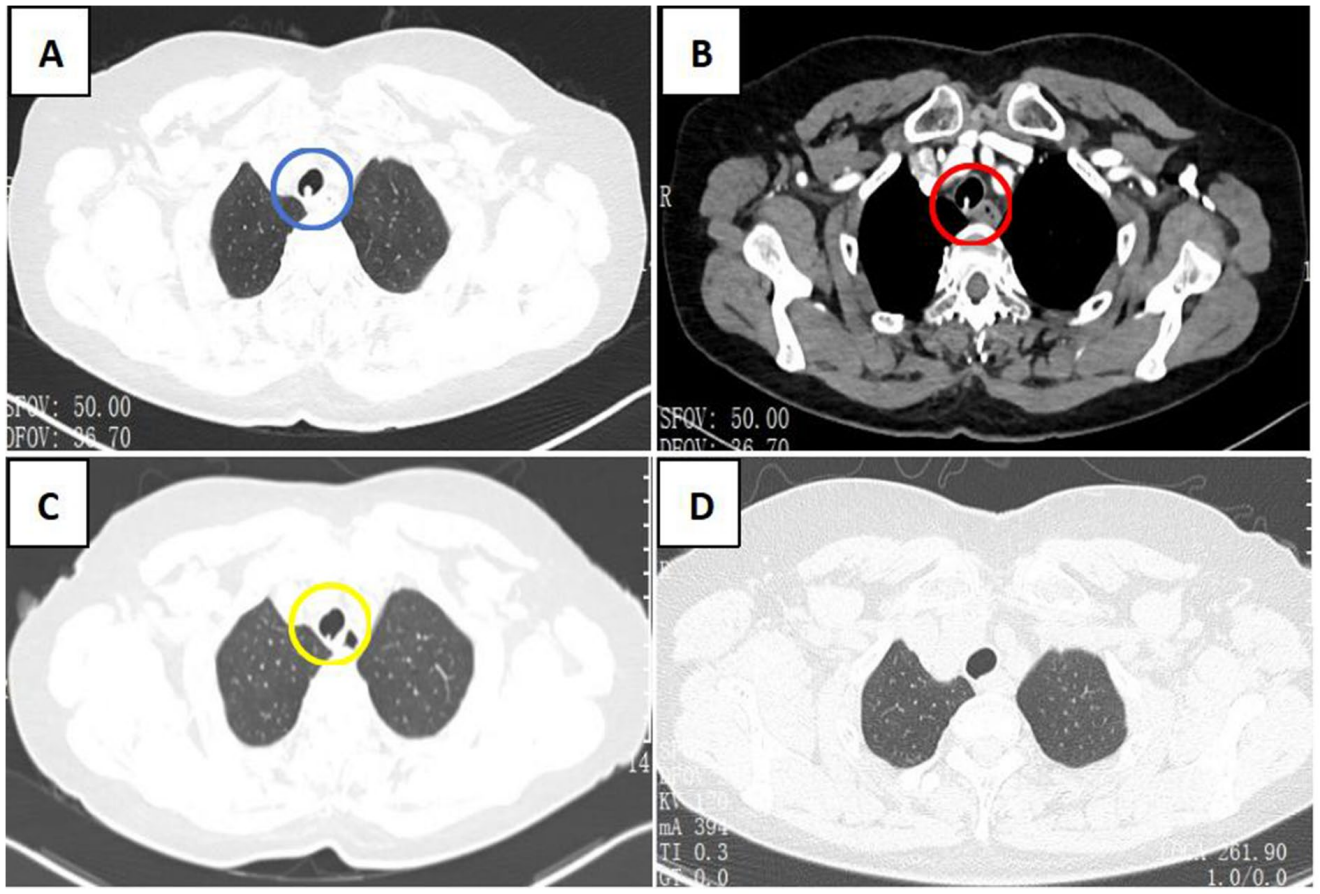
CT scan of the chest. **(A)** CT scan of the chest shows a new spherical organism in the upper tracheal segment with a smooth surface (blue circle). **(B)** CTA scan of the chest shows enhanced nodules in the tracheal, which originated from the branches of the thyrocervical trunk (red circle). **(C,D)** The organism shrank significantly after 1 month (yellow circle) and 4 months, respectively.

**Figure 2:**
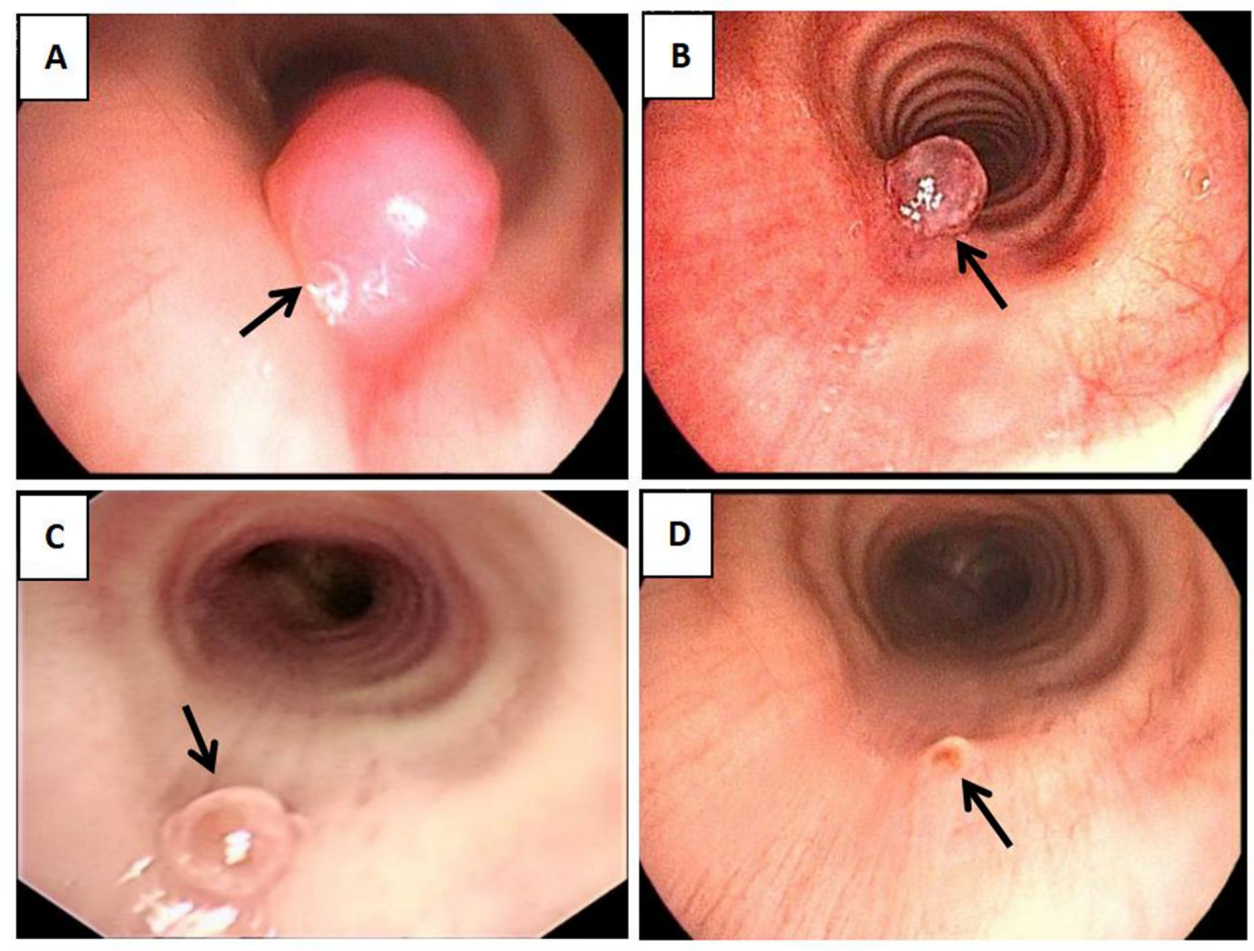
Bronchoscope. **(A)** bronchoscope reveals a smooth spherical neoorganism with pulsation on the right side of the membrane (black arrow). **(B–D)** bronchoscope confirmed that after 3 days, 1 month, and 4 months of treatment. The organism shrank significantly (black arrow).

**Figure 3:**
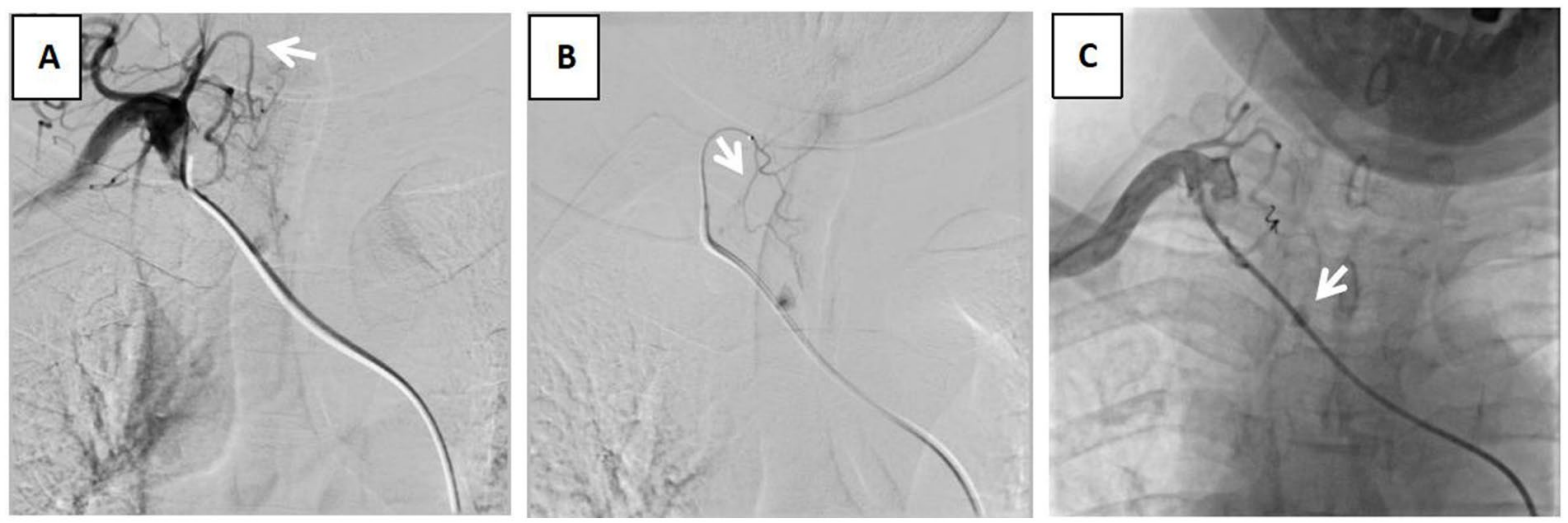
Angiogram. **(A)** preoperative “DAV” catheter angiography shows that the aneurysm vessel originated from the inferior thyroid artery (white arrow). **(B)** Microcatheterization shows that the aneurysm vessel originated from the inferior thyroid artery branch (white arrow). **(C)** After embolization of the microsphere embolized the peripheral vessel and spring coil embolization of the vascular trunk shows contrast retention of the aneurysm (white arrow).

A subsequent CT scan of the chest, CTA of the chest, and bronchoscope were taken next. The chest CT scan showed a new spherical organism in the upper tracheal segment with a smooth surface (Figure 1A). The CTA suggested an aneurysm originating from the branches of the thyrocervical trunk, with a CT value of 334HU (Figure 1B). To assess the condition of the airway, a bronchoscopy was performed, revealing a smooth, spherical neoorganism with pulsation on the right side of the membrane, 5 cm from the glottis (Figure 2A). A subsequent thyrocervical trunk-selective arteriogram demonstrated a pseudoaneurysm arising from the inferior thyroid artery (Figure 3A). The vascular anomaly was embolized using 2-mm detachable micro-coils (Figure 3B). A post-procedure angiogram demonstrated hemorrhage resolution with complete occlusion of the vessel and pseudoaneurysm (Figure 3C). The shrinkage of the pseudoaneurysm was observed after a review at 1 and 4 months later (Figures 1C,D). The change was confirmed through an examination using a bronchoscope (Figures 2B,D).

## Discussion

Pseudoaneurysms of the thyrocervical trunk or its branches are extremely rare. To date, fewer than 50 cases have been reported in the English-language literature. These lesions predominantly arise secondary to traumatic injury or iatrogenic procedures (3, 4). Idiopathic thyrocervical trunk aneurysms exhibit even greater rarity, with only sparse case reports available for reference (5).

Almost all were reported in the form of case reports. The pathogenesis usually involves arterial intimal injury creating discontinuity in the arterial wall, thereby allowing blood into the false arterial lumen, giving rise to pseudoaneurysm formation (6). Presenting symptoms may vary depending on the location and rupture status of the pseudoaneurysm. Complications include stridor, progressive respiratory distress, dyspnea, hemoptysis, and chronic coughs. It can also be expressed as Horner’s syndrome, tingling of the upper extremity, hemothorax, and dysphagia (7, 8).

Although there have been some reports about the pseudoaneurysms of the thyrocervical trunk, the occurrence in the trachea has not been reported. The thyrocervical trunk arises laterally from the vertebral artery and gives origin to the inferior thyroid artery, suprascapular artery, ascending cervical artery, and transverse cervical artery. The diagnosis is typically established through Doppler ultrasonography for superficial lesions and CTA for deep lesions (9). The use of selective angiography is essential for obtaining crucial information on the formation of potential collateral vasculature prior to endovascular intervention (8).

Historically, open surgical repair constituted the standard therapeutic approach, involving proximal/distal vascular control, hematoma evacuation, aneurysm wall excision, and definitive vascular reconstruction (10). Endovascular coiling serves as a viable alternative to open repair and plays a crucial role in surgical candidates who are deemed high-risk. With the development of medicine, coil embolization with micro-coils is a minimally invasive approach and is appropriate even for high-risk patients (11). Ultrasound-guided compression, ultrasound-guided thrombin injections, and liquid embolization with polymerizing agents are also therapeutic options for select patients (12).

We reported the case of a thyrocervical trunk pseudoaneurysm after tracheal intubation, which was discovered after endovascular coiling. Pseudoaneurysm of the thyrocervical trunk is associated with significant mortality, but it can be easily managed via endovascular techniques if detected promptly. To establish a final diagnosis in this case, a systematic exclusion of the underlying causes for the development of the pseudoaneurysm in the patient was required. Pseudoaneurysms are the uncommon result of vascular injury. Most are iatrogenic in origin, although they may rarely develop following blunt trauma. The patient had no history of undergoing central venous catheterization or trauma. The location of the pseudoaneurysm in our case was consistent with the location of the tracheal intubation balloon. We hypothesize that mechanical compression by the overinflated balloon during intubation induced focal arterial wall injury, precipitating pseudoaneurysm formation. To the best of our knowledge, no other case has been reported in the literature. This case underscores the necessity for meticulous technique during tracheal intubation, including controlled balloon inflation pressure and real-time anatomical alignment verification, to mitigate iatrogenic vascular injury.

Pseudoaneurysm of the thyrocervical trunk is associated with significant mortality; however, timely detection allows for effective management through endovascular techniques. This case report aims to raise awareness of this uncommon yet potentially fatal complication.

## Data Availability

The raw data supporting the conclusions of this article will be made available by the authors, without undue reservation.
